# Evaluation of biocontrol efficacy of rhizosphere dwelling bacteria for management of Fusarium wilt and Botrytis gray mold of chickpea

**DOI:** 10.1186/s12863-023-01178-7

**Published:** 2024-01-15

**Authors:** Gurreddi Bhargavi, Meenakshi Arya, Prashant Prakash Jambhulkar, Anshuman Singh, Ajaya Kumar Rout, Bijay Kumar Behera, Sushil Kumar Chaturvedi, Ashok Kumar Singh

**Affiliations:** Rani Lakshmi Bai Central Agricultural University, Jhansi, 284003 Uttar Pradesh India

**Keywords:** Chickpea, Fusarium wilt, Botrytis Gray Mold, Rhizosphere, Bioagents

## Abstract

**Background:**

Chickpea (*Cicer arietinum* L.) production is affected by many biotic factors, among them Fusarium wilt caused by *Fusarium oxysporum* f. sp. *ciceri* and Botrytis gray mold caused by *Botrytis cinerea* led to severe losses. As fungicide application is not advisable, biological management is the best alternative for plant protection. The rhizosphere-dwelling antagonistic bacteria are one of the important successful alternative strategy to manage these diseases of chickpea. Rhizosphere dwelling bacteria serve as biocontrol agents by different mechanisms like producing antibiotics, different enzymes, siderophores against pathogens and thereby reducing the growth of pathogens.

**Results:**

The present study aimed to isolate rhizospheric bacteria from the soils of different chickpea fields to evaluate biocontrol efficacy of the isolated bacteria to manage Fusarium wilt and Botrytis gray mold in chickpea. A total of 67 bacteria were isolated from chickpea rhizosphere from Bundelkhand region of India. Study revealed the isolated bacteria could reduce the *Fusarium oxysporum* f. sp. *ciceris* and *Botrytis cinerea* infection in chickpea between 17.29 and 75.29%. After screening of all the bacteria for their biocontrol efficacy, 13 most promising bacterial isolates were considered for further study out of which, three bacterial isolates (15d, 9c and 14a) have shown the maximum in vitro antagonistic effects against *Fusarium oxysporum* f. sp. *ciceri* and *Botrytis cinerea* comparable to in vivo effects. However, Isolate (15d) showed highest 87.5% and 82.69% reduction in disease against Fusarium wilt and Botrytis gray mold respectively, under pot condition. Three most potential isolates were characterized at molecular level using 16S rRNA gene and found to be *Priestia megaterium* (9c and 14a) and *Serratia marcescens* (15d).

**Conclusion:**

This study identified two native biocontrol agents *Priestia megaterium* and *Serratia marcescens* from the rhizospheric soils of Bundelkhand region of India for control of Fusarium wilt, Botrytis gray mold. In future, efforts should be made to further validate the biocontrol agents in conjugation with nanomaterials for enhancing the synergistic effects in managing the fungal diseases in chickpea. This study will definitely enhance our understanding of these bioagents, and to increase their performance by developing effective formulations, application methods, and integrated strategies.

**Supplementary Information:**

The online version contains supplementary material available at 10.1186/s12863-023-01178-7.

## Background

India is the largest producer and consumer of pulses worldwide and cultivates pulses on 27–28 million hectares throughout 10–12 major states. The chickpea (*Cicer arietinum* L.), among these pulse crops, is a vital crop in the warm temperate and semi-arid regions of the world. It is the cheapest source of protein and makes up the majority of humans’ dietary protein. It is a significant source of vitamins, minerals, fiber, and energy [1, 2]. India is the world’s greatest producer of chickpea, producing 13.63 million tonnes of the crop, but its yield is substantially lower than anticipated because of numerous biotic stresses [2]. The majority of them, including fungi, have an impact on the roots, stems, leaves, flowers, and pods of chickpea. The diseases infecting chickpea can be further classified as soil borne and foliar diseases of which major soil borne fungal diseases of chickpea are Fusarium wilt (*Fusarium oxysporum* f. sp. *ciceri*), Dry root rot (*Rhizoctonia bataticola*), Collar rot (*Sclerotium rolfsii*) and Stem rot (*Sclerotinia sclerotiorum*) while ascochyta blight (*Ascochyta rabiei*), Botrytis gray mold (*Botrytis cinerea*) and Rust (*Uromyces cicer arietini*) are the major foliar diseases. These diseases in chickpea can result in yield losses of up to 100% depending on crop stage and level of infection [3, 4].

Fusarium wilt (FW) is one of the most important soil borne diseases, resulting in major economic losses of between 10% and 40%, however, it has the ability to completely wipe out crops in disease-friendly environments [5]. The fungus, *Fusarium oxysporum* forma specialis (f. sp.) *ciceris* (FOC), which is spread through soil and seeds, is the causal organism for this disease. A soil-borne fungus with putatively pathogenic and non-pathogenic strains, FOC is a member of the Fo species complex. In many agricultural crops, plant pathogenic Fo strains cause cortical rot and vascular wilt. Based on their ability to infect different cultivars of a plant species, they are separated into races and host-specific forms (formae speciales, ff. spp.) [5]. There are numerous strains of Fo in soil, both supposedly non-pathogenic and pathogenic. More than 150 plant species are affected by these pathogenic strains, which have the ability to cause disease.

Based on disease reactions of host differential chickpea cultivars, eight physiological races (0, 1 A, 1B/C, 2, 3, 4, 5, and 6) have been documented globally in Foc [6]. Based on symptoms, there are two pathotypes: one that causes yellowing and the other causes wilting [5, 7]. The wilting symptoms that are caused by races 1 A, 2, 3, 4, 5 and 6 include severe chlorosis, flaccidity, vascular discoloration and plant mortality. Races 0 and 1B/C cause yellowing symptoms and are less virulent than the other races [5]. The eight races are geographically dispersed differently, with race 1 A being more prevalent in India, the Mediterranean region, and California [8]. Races 0, 1B/C, 5 and 6 are primarily found in the USA and the Mediterranean regions, while races 2, 3 and 4 are found in India and Ethiopia [5, 9, 10].

The pathogen was reported with eight races from all over the world [9, 11]. Races 0, 1 A, 1B/C, 5, and 6, have been reported from the United States and Spain and races 1 A, 2, 3, and 4 from India.

Affected plants may cluster in patches or appear to be dispersed throughout the fields [5, 12]. Disease symptoms can be observed at any stage of plant development. Early wilt signs, which include flaccidity of individual leaves followed by a dull-green discoloration, desiccation, and collapse of the entire plant, can appear 25 days after planting in cultivars that are extremely sensitive to the disease. The symptoms can emerge up to the podding stage (referred to as “late wilt”) and are typically more noticeable at the beginning of blooming i.e. 6 to 8 weeks after sowing. Petioles, rachis, and leaflets begin to droop on late wilting plants, which are followed by yellowing and necrosis of the foliage. At first, the upper portion of the plant shows signs of drooping, however after a few days the entire plant collapses. When vertically split or cross-sectioned, the xylem tissues of damaged plants develop a dark-brown discoloration that is noticeable in the roots and stems [5, 10, 12].

Botrytis gray mold (BGM) caused by *Botrytis cinerea* is one of the foliar diseases and has been reported as a significant biotic barrier to the production of chickpea, resulting in yearly yield losses of 10–15% in India and Spain and losses of 70–100% in years with severe outbreaks [13, 14]. BGM is also a serious disease in some areas of Bangladesh, India, Nepal, Pakistan, Australia, and Argentina. However, according to reports from the Gurdaspur belt of Punjab in Northwest India in 2014-15, BGM infection can result in up to a 100% yield loss under favorable conditions. The entire aerial parts of the plant, such as the leaves, flowers, pods, branches, and stems are affected by the fungus. However, the flowers, pods, and growth tips are the most vulnerable to the pathogen [15]. The pathogen can be found in two different forms: *Botryotinia fuckeliana* in the teleomorph stage and *B. cinerea* in the anamorph stage. The crop is susceptible to this disease because of strong and prolonged winter rains, cloudiness, high evening dew, excessive irrigation, early planting, and a dense plant canopy. *B. cinerea* survives well in temperatures between 20 and 25 °C and is most active when temperatures surpass 15 °C. Symptoms caused by the pathogen include powdery gray mold. White fungal growth that eventually turns grey, as a result of the massive spore formulation on the plant part is the characteristic of the fungus infection. The pathogen can produce millions of spores on a single lesion on the stem of a chickpea. The spores of the fungus can be blown over great distances, and if they land on chickpea plants, they can remain dormant till favorable conditions for their growth. The lesions and grey ‘fuzz’ become visible after 5–7 days of the infection under ideal circumstances. The drooping of sensitive terminal branches caused by injury is a typical field indicator. Since the pods are damaged by severe BGM infections, there are either no seeds at all or only tiny and shriveled seeds are obtained [9, 16]. Due to high levels of host tolerance to BGM, this disease causes a potential threat to chickpea crops worldwide.

Over the period, the majority of plant disease management techniques relied on the application of chemical fungicides. However, the repeated and unchecked use of pesticides has a negative knock-on effect on human health, natural soil microbiota, and plant life. Additionally, fungicide-resistant pathogenic fungi have been reported to exist [17–19]. Further, chemical treatment for many crop diseases is also not economical, especially for small scale farmers. Foliar fungicides can be used to control diseases like BGM; however, it could be difficult to manage when the environment is favorable for growth of the disease. Further, seed treatment is also not very effective against BGM. Thus, biological management is the best option for plant protection when environmental and soil health concerns are on the rise. Alternatives to synthetic fungicides that can effectively treat a variety of plant diseases without harming the environment include the rhizosphere-dwelling antagonistic bacteria [20–22] and thus utilizing the antagonistic effect of these rhizosphere dwelling bacteria would be a successful alternative strategy to manage these diseases [23–25]. Bacteria found in the roots of plants can affect their general health as well as their growth and development [25]. Bacteria serve as biocontrol agents by different mechanisms like producing antibiotics, different enzymes, and siderophores against pathogens and thereby reducing the growth of pathogens. By hydrolyzing a number of polymeric substances, lytic enzymes such as chitinase, cellulase, protease, and DNase generated by soil bacteria also directly limit pathogenic activities [26–28]. Furthermore, as fungal siderophores have a lesser affinity for iron that is present in soil, bacterial siderophores play a significant role in suppressing plant diseases through iron sequestration [29, 30]. Further, comprehensive research in the past few decades has highlighted the role of both gram-positive and gram-negative bacterial strains as promising biological control agents [18, 25, 26].

The present study aimed to isolate rhizospheric bacteria from the soils of different chickpea fields and to evaluate biocontrol efficacy of the isolated bacteria to manage Fusarium wilt and BGM of chickpea. Biocontrol experiments were conducted against *Fusarium* wilt and BGM infected chickpea in the lab and experimental pots to assess the efficacy of the potential rhizosphere dwelling bacteria.

## Materials and methods

### Sampling locations

**Fig. 1:**
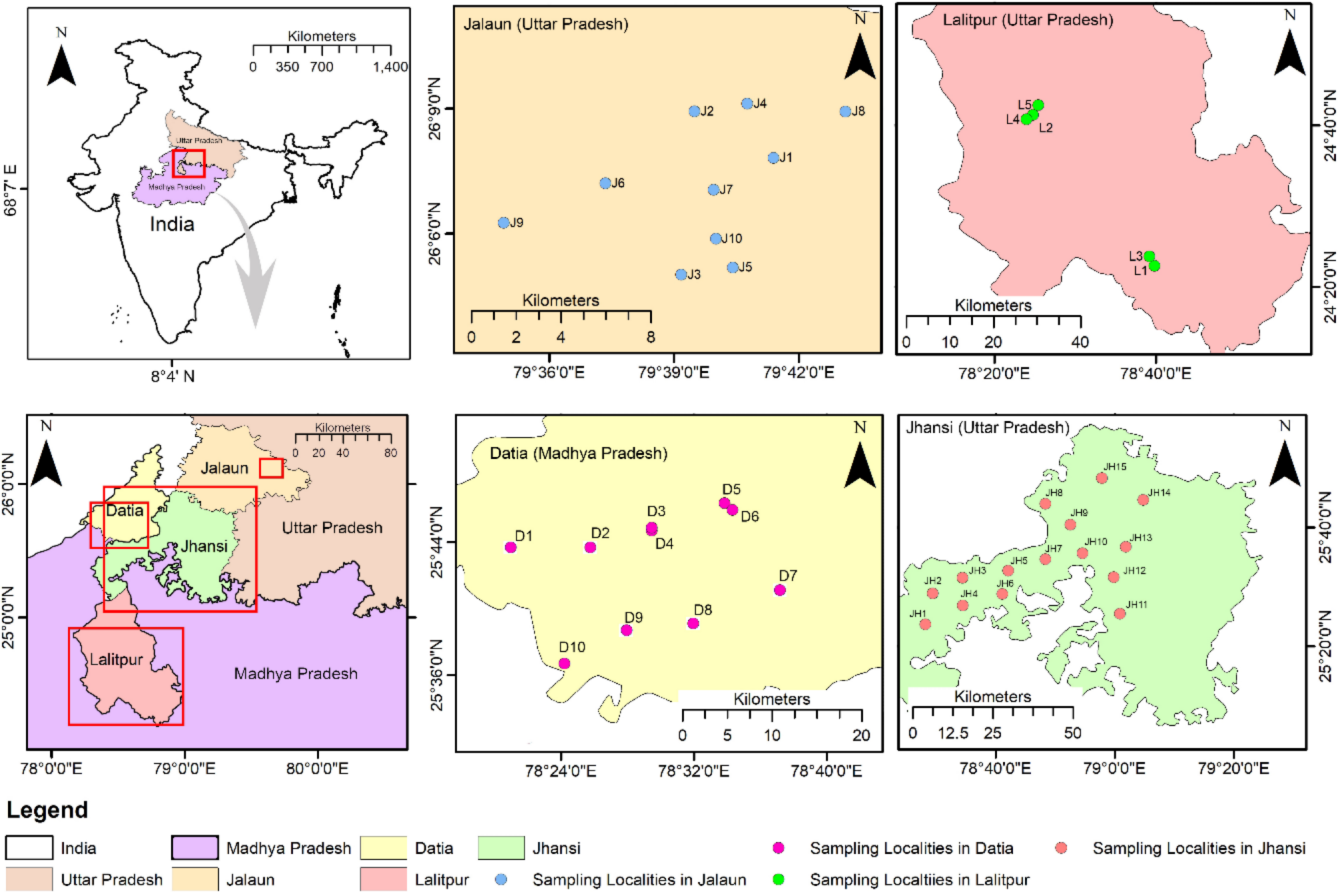
Maps showing rhizospheric soil sampling sites. 40 soil samples were collected from different districts (Jalaun, Lalitpur, Jhansi in Uttar Pradesh and Datia in Madhya Pradesh) of Bundelkhand region in India. The map of soil collection site is prepared using ArcGIS 10.2.1 platform

Forty soil samples were collected from different sites comprising four districts of the Bundelkhand region of India viz. Jhansi (15), Jalaun (10), Lalitpur (5), and Datia (10) (Fig. 1). The sampling sites, represent a region of central India comprising the hilly Vindhyan region and the northeastern plain. It is characterized by variable climatic conditions, intensified by undulating topography, erratic rainfall, areas with degraded forests and rapidly shrinking surface water resources. It is largely rainfed and is perturbed with drought conditions frequent in the region leading to unstable socio-economic conditions and food insecurity.

### Isolation of bacteria from soil sample

To isolate the root associated bacteria, 40 soil samples from the rhizosphere of chickpea fields were collected during rabi 2020–2021 from four districts in the Bundelkhand region. The chickpea plants and the associated soil samples were collected in the crop growing season at a depth of 20–25 cm. The samples were transported safely in sterilized polythene packs to the laboratory and were kept in the 4^o^C freezer until they were used for bacterial isolation. The soil from rhizosphere was later on collected by gentle shaking off the soil that is adhered to roots. 1 g of soil was then placed into 9 mL autoclaved water and with regular shaking during 1 h [31]. By employing the serial dilution approach and using different nutrient media viz. Nutrient Agar, King’s B, Pseudomonas agar (HIMEDIA) and Trypticase Soy Agar were procured from HIMEDIA, bacterial colonies were obtained from the collected soil samples. The first tube was filled with one gm of soil, which was then serially diluted up to 10^6^dilutions. One ml of this dilution was used for spreading on nutrient media using glass L-shaped spreader to obtain the targeted bacterial colonies. The plates were incubated at 25 °C for 24 h in the dark and were observed for microbial growth. The single colonies having different morphological characteristics (shape, size, colour, margins, opacity and appearance) were selected for sub-culturing. The pure culture was obtained after incubation at 25 °C for 24 h in the dark. The isolates of the bacteria thus obtained were labeled properly for further use.

### Pathogen and pathogenicity test

The culture of FOC was isolated from the wilt infected chickpea plants, collected from fields of Rani Lakshmi Bai Central Agricultural University, Jhansi, India, on Potato Dextrose Agar (PDA) media and the pure culture was obtained by the hyphal tip method. While the pure culture of *B. cinerea* was procured from ICAR-Indian Institute of Pulse Research, Kanpur, India. The FOC was grown on PDA and was used for mass culture on chickpea grains for soil inoculation. The chickpea grains were soaked overnight and were placed in conical flasks with a capacity of 500 ml then autoclaved twice at 15 kg/cm pressure for 15–20 min at 120 °C. The seeds were then infected with mycelial discs (3 mm in diameter) of FOC and cultured for 7 to 10 days at 25 °C in a BOD incubator. The soil was made sick by infesting the sterilized soil with 10^6^ CFU of FOC per g soil as followed by [32].

Similarly, for mass multiplication of *B. cinerea*, the fungus was grown on potato dextrose broth in 500 ml conical flasks for seed and foliar inoculation. The sterilized broth was inoculated with a culture of *B. cinerea* which was then cultured for two weeks at 25 °C. To produce the fungus suspension for foliar spray, the mat was ground in a sterilized jar of mixer grinder with sterilized distilled water. Using the dilution plate approach, the colony-forming units (CFU)/ml suspension was calculated. By adding sterilized distilled water, the fungal suspension was standardized to maintain 10^5^ CFU/ml of *B. cinerea*. Each plant received 5 ml of the suspension, which contained 5 × 10^5 ^CFU/ml in actual count.

Both pathogens were also tested for their pathogenicity before assessing the antagonistic effect of rhizobacteria. Artificial inoculation of FOC was done by multiplying chickpea grains and mixing them with sterile soil in the ratio of 1:9 [inoculum: soil (w/w)] to make the soil sick. The pots were left undisturbed for 4–5 days for the fungus to grow and stabilize in the soil. The seeds of susceptible cultivar viz. JG-62 was sown in it. Similarly, spore suspension of *B. cinerea* with 3 × 10^5^ CFU/ml was sprayed on a susceptible variety of chickpea plants (JG-62) sown in sterilized soil in pots. Ambient temperature (25 °C) and humidity (95%) were maintained for the development of *B. cinerea* as fungi thrive well in humid atmospheres. Control pots were not inoculated with the pathogen in both cases. Disease symptoms were recorded after every two days for FOC infected chickpea and every day in the case of BGM infected chickpea. Upon development of the symptoms, the pathogens were re-isolated and compared with the original cultures for confirmation of pathogenicity.

### Morphological characterization of the bacterial isolates

The phenotypic traits of all the isolated bacteria were examined based on the biochemical and morphological characteristics. The cultural characteristics such as shape, size, color, margin, and appearance of the colony of 5 days old bacterial culture were examined.

### Test for in-vitro antagonism of the isolated bacteria against FOC and ***B. cinerea***

The presence of antagonistic activity against FOC and *B. cinerea* was initially assessed in vitro for all isolated rhizobacteria using the dual culture method on PDA media [33]. A mycelial disc (5 mm diameter) of the seven days old cultures of the fungal pathogen was taken from advancing zone of hyphae growing in already inoculated PDA plates with a pure culture of the fungus. The disc was then carefully kept at the centre of a Petri plate (90 mm diameter) containing freshly prepared PDA media. The 24 h. old bacterial culture was then streaked at parallel, 3 cm away on both sides of the fungal disc. The plates were incubated at 28 °C and checked daily for inhibition until the fungal growth on the control plate (inoculated only with the pathogen) reached the edge of the plate. Inhibition of fungal growth along the line of streaking of bacteria indicated antagonistic activity of the isolated bacterial strain. The selected isolates were re-tested for antagonism against both pathogens following the same method. Percentage inhibition of the mycelial growth of test pathogens was calculated as [(Colony Diameter of pathogen growth in control plate (mm)- Colony Diameter of pathogen growth in presence of antagonist (mm)/ Colony Diameter of pathogen growth in control plate (mm)] X 100 [34]. The dual culture tests were performed in three replications and the data was averaged.

### Biochemical characterization of the bacterial isolates

Thirteen bacterial isolates showing ≥ 51% inhibition of both the pathogens in dual culture technique were selected from *in-vitro* screening and were subjected to different biochemical tests such as KOH, amylase, catalase, citrate utilization, indole production, methyl red, Voges Prausker’s and oxidase test using standard protocols [35]. The results of these tests were scored as either positive or negative.

### Screening for bacterial isolates for other plant growth promoting (PGP) factors and antimicrobial secondary metabolites production

The isolated promising bacterial isolates with the highest inhibition percentages for both FOC and BGM were selected and checked for their PGP factors and other antimicrobial secondary metabolites. The following tests were conducted in three replicates for the screening of bacterial isolates.

### Cellulase activity

Apart from biocontrol properties, cellulase has plant growth promoting traits as well [36]. The qualitative assessment of the production of cellulase by the potential bacterial isolates was performed by the method proposed by [37, 38]. Bacteria were inoculated on CMC agar media and the agar media was flooded for 15 min with an aqueous solution of congo red (1% w/v) after incubation. After removing the congo red solution, the plates were further treated with 1 M NaCl by flooding with it and left for 15 min. Isolates were considered cellulase-producing if a halo was formed around the colony. Isolates with halos less than 1.0 mm were considered weak producers (+). Isolates with halos greater than 1.0 mm were considered strong producers (++). Non-producers (-) were indicated by the absence of a halo [39].

### Ammonia production

Ammonia production by bacterial isolates was determined using Nessler’s reagent. Bacterial isolates were examined for production of ammonia in peptone broth. Freshly developed cultures of each bacterium were inoculated separately in 10 mL peptone water and incubated for 48–78 h at 28 ± 2 °C along with control [40]. 0.5 mL of Nessler’s reagent was then added to each tube after incubation. The development of brown to yellow colour was observed indicating production of Ammonia [41].

### Hydrogen cyanide production (HCN)

Test for HCN production was carried out by the method as described by [42]. A sterilized nutrient broth amended with glycine (4.4 g/l), already poured into the sterile test tubes, was inoculated by 24 h old bacterial culture. The filter paper (Whatman No. 1) was cut into strips and immersed in 0.5% picric acid. These strips were inserted between the plug and the inner wall of the test tube so that it hung above the inoculated broth. Inoculated tubes were sealed with parafilm tape to hold the gaseous metabolic produced by the antagonistic bacteria and allow for a chemical reaction with picric acid on the top. After incubation for a week at 28 °C, the colour of the filter paper was observed. The positive result was indicated by the color shift of a filter paper strip immersed in picric acid from yellow to red.

### Siderophore production

Siderophore production was determined using the Universal Chromazurol S (CAS) assay described by [43] and modified by [44]. The log-phase bacterial culture was spread on nutrient agar plates amended with the CAS solution. The plates were incubated at 28 °C under dark conditions for 3–5 days. The appearance of yellow to orange zones confirms siderophore production.

### Indole acetic acid (IAA) production

Production of the phytohormone IAA was investigated spectrophotometrically (JENWAY®) by using the method of [45]. Bacterial cultures were grown for 48 h in nutrient broth (NB) at 28 ± 2 °C to test the bacterial isolates for the production of IAA. 100 μl each of all the bacterial suspensions of fully-grown bacterial culture was inoculated in 5 ml Nutrient Broth (NB) media separately both in absence and presence of 500 μg/ml of tryptophan. Later, it was placed for 48 h in an incubating shaker at 28 ± 2 °C. Centrifugation of bacterial culture was done at 3000 rpm for 15 min and the supernatant (2 ml) was mixed with two drops of ortho-phosphoric acid and 4 ml of the Salkowski reagent (50 ml, 35% of perchloric acid, 1 ml 0.5 M FeCl_3_ solution). The absorbance maxima of IAA (development of pinkish colour) in the bacterial culture supernatant were recorded at 530 nm.

### Test for the efficacy of antagonistic bacteria under in vivo conditions against FOC and ***B. cinerea***

#### Evaluation of bacteria against FOC

The pots (25 cm diameter) were filled with FOC sick soil (as described previously). Ten seeds each of susceptible (JG-62) and resistant genotypes (WR-315) were used in the experiment. Further, based on results of antagonistic effect by dual culture technique, only eight identified promising bacteria were used for this experiment. The experiment was planned in three replications. JG 62 and WR 315 seeds were treated with eight different bacteria by soaking the seeds in bacterial suspension (10^8 ^CFU/ml) in all the replications. Seeds without bacterial treatment were sown in control pots and the germination of the seeds in each pots was observed. Ten plants per pot were uniformly maintained in each pot. Plants were regularly monitored for disease development and the disease reactions were assessed by the percent wilt using the scale as described by [46] and the incidence of wilt disease and percent protection by bacterial isolates were recorded and calculated as per the formula given by [46] and [25].

$${\rm{Per}}\,{\rm{cent}}\,{\rm{Incidence = }}\frac{{{\rm{Total}}\,{\rm{number}}\,{\rm{of}}\,{\rm{infected}}\,{\rm{plants}}}}{{{\rm{Total}}\,{\rm{number}}\,{\rm{of}}\,{\rm{plants}}}}{\rm{ \times 100}}$$

$${\rm{Percentage}}\,{\rm{of}}\,{\rm{protection = }}\frac{{{\rm{A}} - {\rm{B}}}}{{\rm{A}}} \times 100$$

Where A = % incidence in pathogen inoculated control plants; B = % incidence in treated plants.

#### Evaluation of bacteria against ***B. cinerea***

Ten (10) surface sterilized seeds each of susceptible (JG 62) and tolerant genotypes (PBG 7, DCP 93 − 2) were sown separately in pots containing sterilized soil in three replications. Seedlings were then allowed to grow in the specifically designed chamber for BGM disease development where the temperature was maintained at 25 °C and the light hours were controlled by using lights. 15 days old seedlings were sprayed with ten different bacterial treatments (10^8^ CFU/ml) based on results of antagonistic effect under in vitro conditions. The non-inoculated pots of each genotype served as a control. After a spray of the bacterial isolate, the spore suspension of *B. cinerea* was sprayed on the seedlings and the pots were properly covered with polythene bags to maintain the required humidity (95%). The development of disease was observed and severity was recorded by selecting three plants randomly from each treatment from all replications. The plants were scored individually using a 1–9 disease rating scale [47], where: 1 = asymptomatic (Free), 3 = resistant (R), 5 = moderately resistant (MR), 7 = susceptible (S), and 9 = highly susceptible (HS). Percent Disease Index (PDI) was calculated using the following formula [48].

$${\rm{PDI = }}\frac{{{\rm{Sum}}\,{\rm{of}}\,{\rm{all}}\,{\rm{ratings}}}}{{{\rm{Total}}\,{\rm{number}}\,{\rm{of}}\,{\rm{ratings}} \times {\rm{Maximum}}\,{\rm{disease}}\,{\rm{grade}}}} \times 100$$

### Molecular characterization of isolated bacteria

Based on in vitro and *in-vivo* studies, three promising antagonistic bacteria isolates were selected and were subjected to molecular characterization using the 16S rRNA gene sequence analysis. The total genomic DNA of the bacteria was extracted by CTAB method [49, 50]. PCR was performed using primers 27 F (5^/^- AGAGTTTGATCMTGGCTCAG-3^/^) and 1492R (5^/^- TACGGYTACCTTGTTACGACTT-3^/^) [51] in a reaction mixture containing 4 μl of template DNA, 22 μl of PCR Master mix, 1 μl each of forward and reverse primers and 22 μlsterile distilled water to make up the 50 μl reaction volume. The reaction mixture was kept in 96 well thermal cycler (Veriti®) and the conditions for PCR were initial denaturation temperature at 94 °C for 4 min, 35 cycles of denaturation at 94 °C for 1 min, annealing at 52.5 °C for 45 s., extension at 72 °C for 1 min and final extension for 10 min at 72 °C. The amplification products were separated by electrophoresis on a 1.2% agarose gel after staining with ethidium bromide (0.5 μg mL^− 1^). Fragments were visualized and photographed under UV using the Gel doc system from Syngene®. The amplified products were sequenced [52] at M/S MEDAUXIN, Bengaluru, India. The sequences were subjected to the BLAST search program of the NCBI, for a similarity search of the obtained sequence with available 16S rRNA nucleotide sequences of related species available in GenBank NCBI. The sequences of the bacterial isolates having similarity ranging from 98 to 100% with the target sequence were used for sequence alignment. All the sequences were aligned using the CLUSTAL W multiple sequence alignment program. Phylogenetic trees were constructed by using Mega 11 software using the Nabour-joining method with a 1000 bootstrap value [53].

### Statistical analysis

To evaluate the significance between treatments, the results were statistically processed through the Web Based Agricultural Statistical Software Package (WASP 2.0) developed by ICAR-Central Coastal Agricultural Research Institute, Ela Goa, India.

## Results

### Isolation of bacteria

A total of 67 bacterial isolates were isolated from 40 soil samples collected from four districts of Bundelkhand region of India viz., Jhansi, Datia, Lalitpur and Jalaun by using dilution plate method (Table 1).

**Table 1:** List of antagonistic bacterial isolates identified from different rhizosphere soil samples from Bundelkhand region of India

Soil sample collection sites	Code assigned	Total no. of Bacteria	Antagonistic Bacterial ID
Rajapur, Jhansi	1	1	-
Raksha, Jhansi	3	1	-
Ambabai, Jhansi	6	4	6a, 6c
Hastinapur, Jhansi	8	3	8b, 8c
Babina, Jhansi	9	4	9c
Dhikauli, Jhansi	10	5	10b, 10c
Imliya, Jhansi	12	3	12a, 12b
Mankua, Jhansi	13	2	-
University Campus, Jhansi	14	3	14a
Bhojla, Jhansi	15	4	15c, 15d
Noner, Datia	16	3	16a
Bhander, Datia	18	5	18a, 18e
Chirula, Datia	19	2	19a
Hasanpur, Datia	21	3	21c
Govindnagar, Datia	22	5	22a, 22b, 22d
Nagra, Jalaun	26	4	-
Jalaun, Jalaun	27	4	27a
Singpura, Jalaun	33	1	33a
Alipur, Jalaun	35	1	-
Pali, Lalitpur	36	2	-
Khaikhera, Lalitpur	37	2	37a
Simmardha, Lalitpur	38	1	-
Lalitpur, Lalitpur	40	3	40a, 40b, 40c

### Pathogenicity test of FOC and ***B. cinerea***

Both pathogens were also tested for their pathogenicity before assessing the antagonistic effect of rhizobacteria. The pathogens were re-isolated and the fresh isolates resembled the original one based on morphology. The pathogenicity test was further confirmed by mortality of the plants grown in FOC infested soil as well as the plant inoculated by *B. cinerea* suspension where in both cases the plants showed more than 50% mortality. However, the plants in the control pots did not show any clinical symptoms.

### Morphological characterization of the bacterial isolates

The morphological characters of the bacterial colonies were recorded and it was observed that the isolates showed varied morphological characters w.r.t. shape, size, colour, margins, opacity and appearance (Table S1). The bacterial colonies were examined for different morphological characters as per standard procedures described by [54]. Based on the morphological characters, all 67 isolates were grouped in seven clusters as described in Table 1.

### Test for in-vitro antagonism of the isolated bacteria against FOC and ***B. cinerea***

**Fig. 2:**
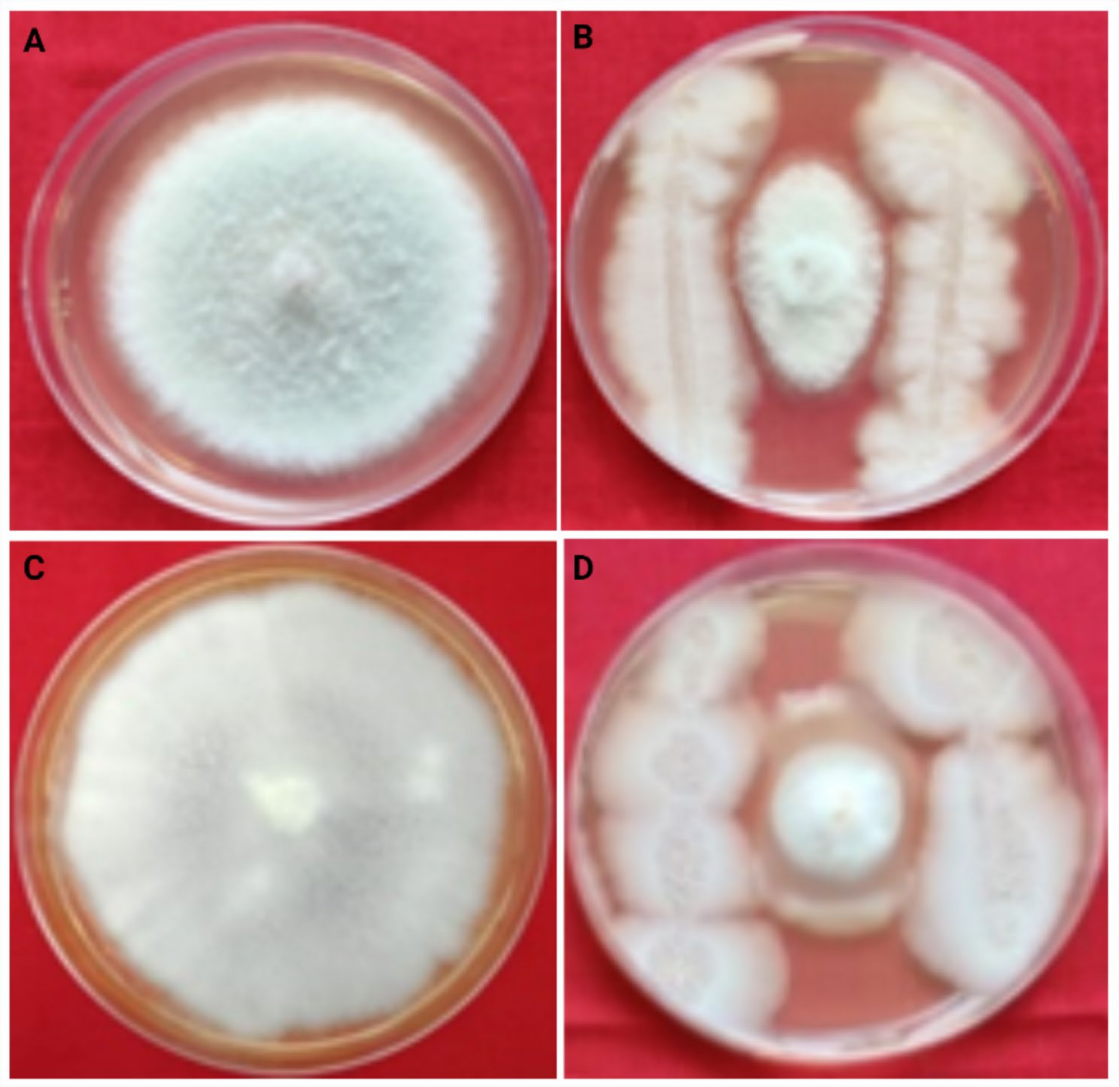
In vitro antagonism of FOC and *B. cinerea* by the identified most potential bacterial isolate (15 d). Control plate of FOC (**A**); plate showing inhibition of FOC by bacterial antagonism (**B**); control plate of *B. cinerea* (**C**); plate showing inhibition of *B. cinerea* by bacterial antagonism (**D**)

The 67 bacterial isolates were isolated from the rhizosphere of chickpea and were evaluated for their antagonistic activities against FOC and *B. cinerea* isolates in vitro. The results of dual culture assay revealed that, out of 67 tested isolates, only 25 isolates showed the antagonism with percentage inhibition ranging from 17.29 to 75.29 against FOC and *B. cinerea*. The growth of FOC was significantly reduced in the dual plate assay with > 50% average inhibition by only 10 isolates. On the other hand, 23 isolates were identified as promising for having an antagonist effect against *B. cinerea* inhibiting the growth of the pathogen up to > 50% (Table 2). The maximum percentage of inhibition against FOC was observed upto 67.84% by the bacterial isolate, 15d followed by 14a, 08c, 40a with 67.45, 66.67, 65.10, mean percentage of inhibition respectively (Fig. 2). Similarly, the isolate 15d has shown a maximum inhibition percentage of 75.29% followed by isolate 09c, 15c with 74.12% each (Fig. 2) against *B. cinerea*. On the basis of results of in-vitro screening of 67 isolates, eight most potential isolates viz. 15d, 14a, 8c, 40a, 8b, 40c, 22a, and 9c were selected for evaluation of antagonism against FOC in pot culture experiment. Similarly, ten most potential isolates viz. 15d, 09c, 15c, 08c, 10b, 14a, 06a, 40b, 22a, 10c were selected for in vitro evaluation of these isolates against BGM. Therefore, out of 67 isolates, only 13promising bacterial isolates which include, five isolates showing antagonism against both the pathogens, three that were found effective only against FOC and five isolates that showed antagonism against only *B. cinerea* were considered for further screening. The statistical analysis revealed that the results in dual plate assay for both pathogens (FOC and *B. cinerea*) were highly significant (*p* < 0.05).

**Table 2:** Percentage inhibition of FOC and *B. cinerea* by bacterial isolates

Sl. No.	Antagonistic Bacterial ID	Percentage inhibition of mycelial growth	
		FOC	*B. cinerea*
1.	15d	67.84 (55.46) ^a^	75.29 (60.19) ^a^
2.	9c	58.43(49.85) ^c^	74.12 (59.42) ^a^
3.	15c	-	74.12 (59.42) ^a^
4.	8c	66.67 (54.73) ^a^	67.45 (55.22) ^b^
5.	10b	-	65.10 (53.79) ^b^
6.	14a	67.45(55.21) ^a^	59.22 (50.31) ^c^
7.	40b	-	58.82 (50.08) ^cd^
8.	6a	-	58.43 (49.85) ^cde^
9.	22a	58.43(49.85) ^c^	58.04 (49.62) ^cdef^
10.	10c	-	56.47 (48.72) ^defg^
11.	37a	-	56.08 (48.49) ^efg^
12.	40a	65.10(53.79) ^b^	56.08 (48.49) ^efg^
13.	12b	-	55.69 (48.27) ^fg^
14.	18a	-	54.12 (47.36) ^gh^
15.	16a	-	52.94 (46.68) ^hi^
16.	33a	46.27(42.86) ^e^	51.37 (45.79) ^i^
17.	12a	-	48.63 (44.21) ^j^
18.	27a	-	47.84 (43.77) ^j^
19.	18e	-	41.57 (40.15) ^k^
20.	40c	59.22(50.32) ^c^	36.47 (37.15) ^l^
21.	22d	-	36.08 (36.92) ^l^
22.	6c	-	36.08 (36.91) ^l^
23.	19a	-	30.59 (33.57) ^m^
24.	21c	-	27.84 (31.84) ^n^
25.	22b	52.55(46.46) ^d^	17.25 (24.54) ^op^
26.	8b	64.71(53.55) ^b^	-
	Control	-	-
	CD @ 5%	0.75	1.49
	CV	0.87	1.98

The values in the parantheses are Arc sine transformed values. Values in the same column followed by the same letters are not significantly different at *p* < 0.05

### Biochemical characterization of the selected antagonist bacteria

All selected 13 potential bacterial isolates were subjected to various biochemical assays. It was observed that all the isolates showed a positive response to catalase and sulphide whereas were negative to indole and methyl red test. However, four isolates were found positive and nine were negative for KOH. Similarly, for amylase test eight isolates were found positive and four were negative. Five isolates turned out to be negative and remaining were positive for the oxidase test. The isolates 08c and 14a showed Voges Proskauer’s positive traits, and others were recorded as negative. All the isolates were found to be of motile nature except 8b, 10b, 40a and 40c. Five isolates showed a positive response to citrate utilization while remaining eight isolates turned out to show a negative response. Thus, results revealed that, the isolated bacteria are belong to *Enterobacteriaceae, Bacillaceae* and *Pseudomonadaceae* family (Table S2).

### Screening for bacterial isolates for PGP factors and antimicrobial secondary metabolites production

Additionally, all these 13 isolates were also biochemically characterized to know the various mechanisms like production of hydrolytic enzymes, secondary metabolites, etc., by the bacteria to control pathogens. All isolates except 06a, 22a and 40b were found to be positive for cellulase production and exhibited the highest activity in 08b, 08c, 15c, 15d and 40c. Only four isolates 08b, 08c, 10c and 14a showed positive results for ammonia production and rest were found to be negative for the test. Isolates namely 06 a, 9c, 14a and 15d were found positive for the production of siderophores, while others showed negative results for the test. All isolates except 14a were positive for IAA with the highest production in 06c, 10c, 15c, 40a and 40b. Likewise, all the isolates were found negative for the production of HCN (Table S3).

### Test for the efficacy of antagonistic bacteria under in vivo conditions against FOC and ***B. cinerea***

#### Evaluation of bacteria against FOC

**Fig. 3:**
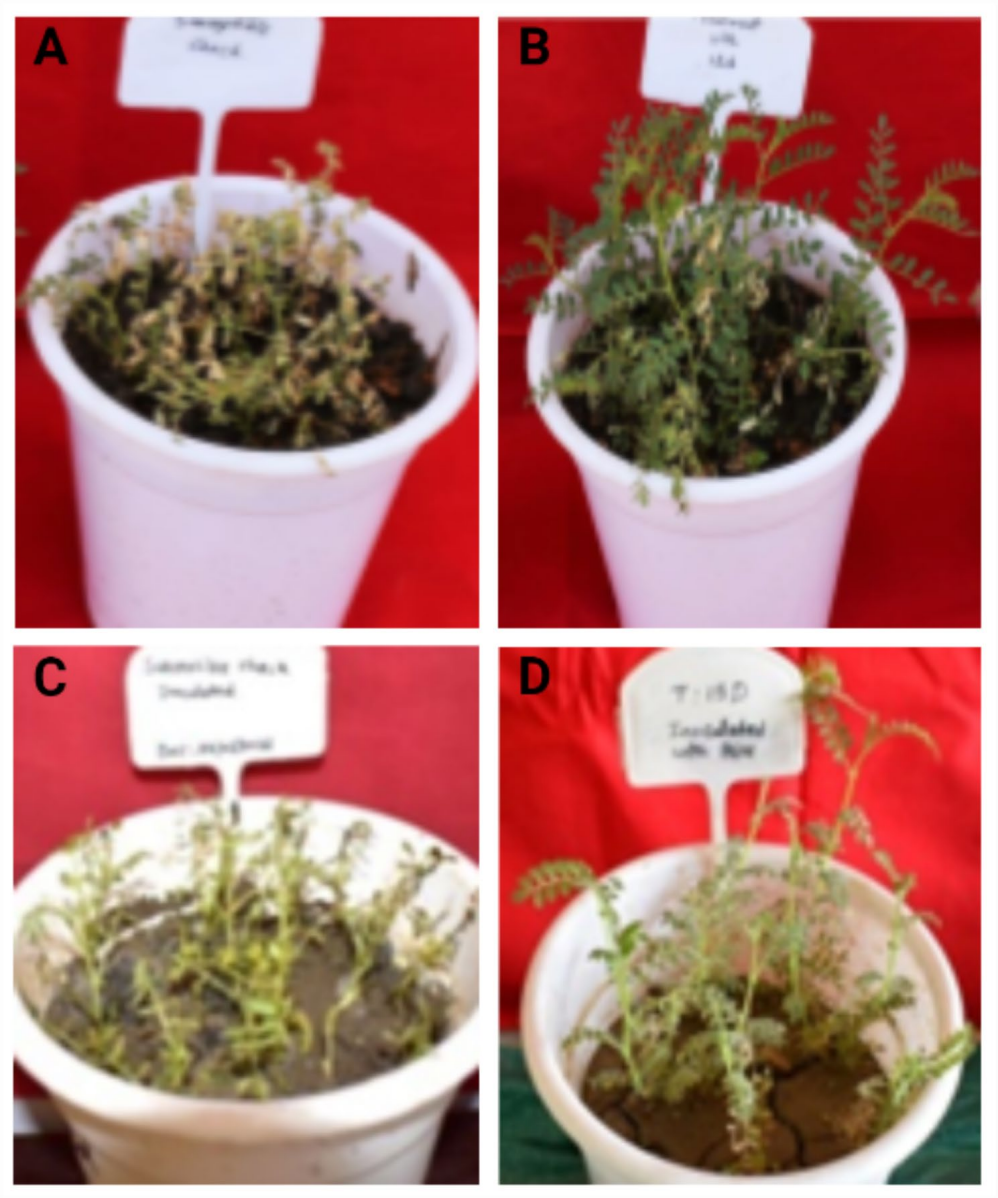
In vivo effect of the identified most potential bacterial isolate (15 d) in managing *Fusarium* wilt and Botrytis gray mold disease. Control pot of FOC (**A**); treatment with potential bacteria showing control of *Fusarium* wilt disease (**B**); control pot of *B. cinerea* (**C**); treatment with potential bacteria showing control of Botrytis gray mold disease (**D**)

Chickpea plants grown in wilt sick soil showed maximum disease reduction by treatment with isolate 15d (Fig. 3). The percent disease incidence using 15d was recorded as low as 11.67 with an overall reduction in the disease to an extent of 87.50 followed by treatment with 14a showing 18.33% disease incidence and reduction of disease up to 80.36% over control. The disease suppression by the different isolates ranged from 21.43 to 87.5% (Table 3).

**Table 3:** Percentage ofdisease incidence and protection by antagonistic bacteria against *Fusarium* wilt of chickpea

Sl. No.	Antagonistic Bacterial ID	Fusarium wilt		
		Disease incidence (%)	Protection (%)	
1	15d	11.67 (19.88) ^g^	87.50	
2	14a	18.33 (25) ^g^	80.36	
3	8c	21.67 (27.21) ^fg^	76.78	
4	40a	31.67 (34.14) ^ef^	66.07	
5	8b	45.00 (42.13) ^de^	51.78	
6	40c	58.33 (49.83) ^cd^	37.50	
7	22a	60.00 (50.86) ^c^	35.71	
8	9c	73.33 (59.00) ^b^	21.43	
9	Control	93.33 (81.86) ^a^	0.00	
	CD (*p* = 0.05)	8.12		
	CV	10.92		

The values in the parantheses are Arc sine transformed values. Values in the same column followed by the same letters are not significantly different at *p* < 0.05

#### Evaluation of bacteria against ***B. cinerea***

The BGM inoculated chickpea plants were treated with ten different potential rhizobacteria and it was observed that the treatment with isolate 15d could control the BGM disease much more effectively with a mean PDI of 16.67% with 82.69% reduction in disease over control (Fig. 3) followed by 09c with 20.37% mean PDI indicating 78.85% disease reduction over control. Remaining, all the potential bacterial isolates were found to minimize the disease ranging from 42.31 to 82.69% (Table 4). The results of in vivo studies were in accordance with the *in-vitro* studies with respect to isolate 15d, 9c and 14a which could effectively control both diseases by more than 65%.

**Table 4:** Percent Disease Index (PDI) and Percent of protection by antagonistic bacteria against Botrytis gray mold disease

S. No.	Antagonistic Bacterial ID	Botrytis Gray Mold		
		Percent Disease Index	Percent of protection	
1	15d	16.67 (23.59) ^h^	82.69	
2	9c	20.37 (26.37) ^gh^	78.85	
3	15c	25.93 (30.58) ^fg^	73.08	
4	8c	31.49 (34.45) ^ef^	67.31	
5	10b	38.89 (38.87) ^de^	59.62	
6	14a	42.59 (41.75) ^cd^	55.77	
7	6a	46.30 (43.92) ^bcd^	51.92	
8	40b	48.15 (45.34) ^bcd^	50.00	
9	22a	51.85 (46.78) ^bc^	46.16	
10	10c	55.56 (48.91) ^b^	42.31	
11	control	96.30 (78.91) ^a^	0.00	
	CD (*p* = 0.05)	6.63		
	CV	9.37		

The values in the parentheses are Arc sine transformed values. Values in the same column followed by the same letters are not significantly different at *p* < 0.05

### Molecular characterization of isolated antagonistic bacteria

Three most promising bacterial isolates i.e., 15d, 09c, 14a that showed maximum disease suppression by inhibition of both the pathogens under *in-vitro* and in vivo studies were considered for molecular characterization. The PCR amplification of 16S rRNA gene yielded 1445 bp amplicon and the PCR product was sequenced. The sequences obtained for bacterial isolates, 15d, 09c and 14a were compared with other sequences using BLAST. The search was limited to sequences from type material, and the identification was based on homology percentage with the reference sequences.

Phylogenetic trees were generated by the neighbor-joining method using the Mega 11 program with Bootstrap values based on 1000 replications [55]. Sequences that showed highest similarity percentages in BLAST homology analysis were selected and used for the construction of the trees. Comparative 16S rRNA gene sequence analysis has been used for bacterial identification at the genus and species levels as well as for inferring phylogenetic relationships.

**Fig. 4:**
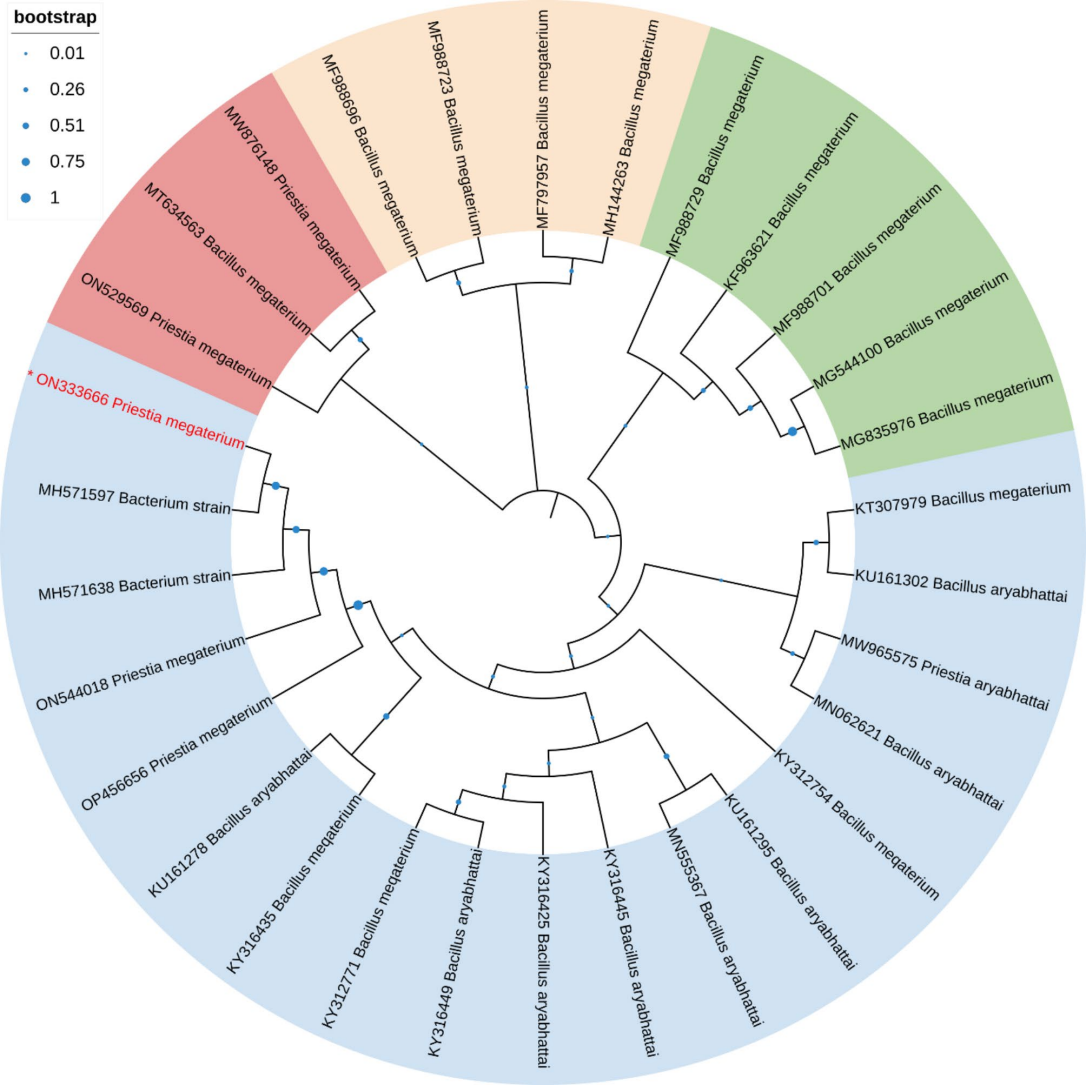
The Neighbor-Joining approach was used to infer the evolutionary history. The evolutionary history of the taxa investigated is represented by the bootstrap consensus tree, which is derived from 1000 replicates. The Maximum Composite Likelihood approach was utilized to calculate the evolutionary distances, which are expressed in the units of base substitutions per site

**Fig. 5:**
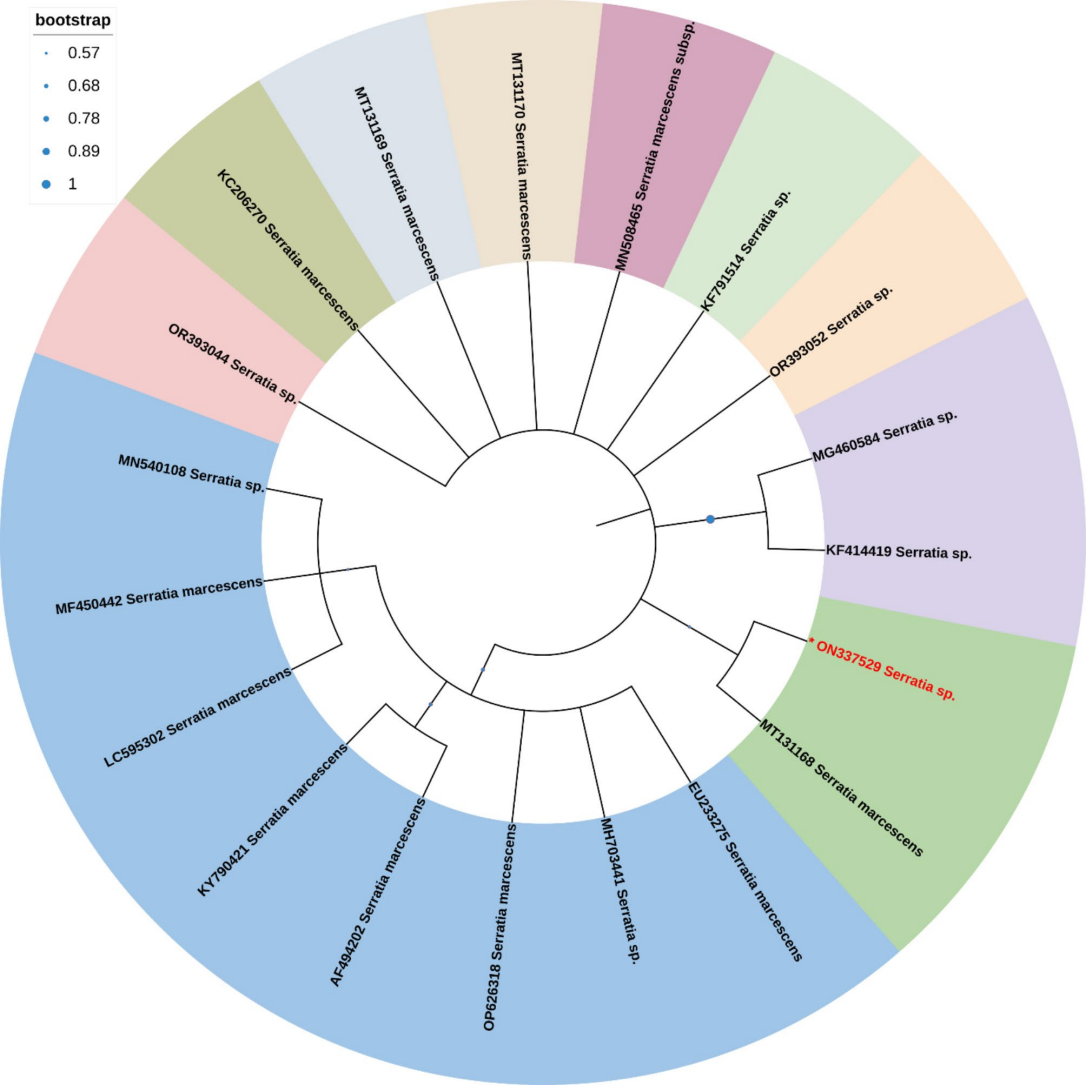
The Neighbor-Joining approach was used to infer the evolutionary history. The evolutionary history of the taxa investigated is represented by the bootstrap consensus tree, which is derived from 1000 replicates. The Maximum Composite Likelihood approach was utilized to calculate the evolutionary distances, which are expressed in the units of base substitutions per site

**Fig. 6:**
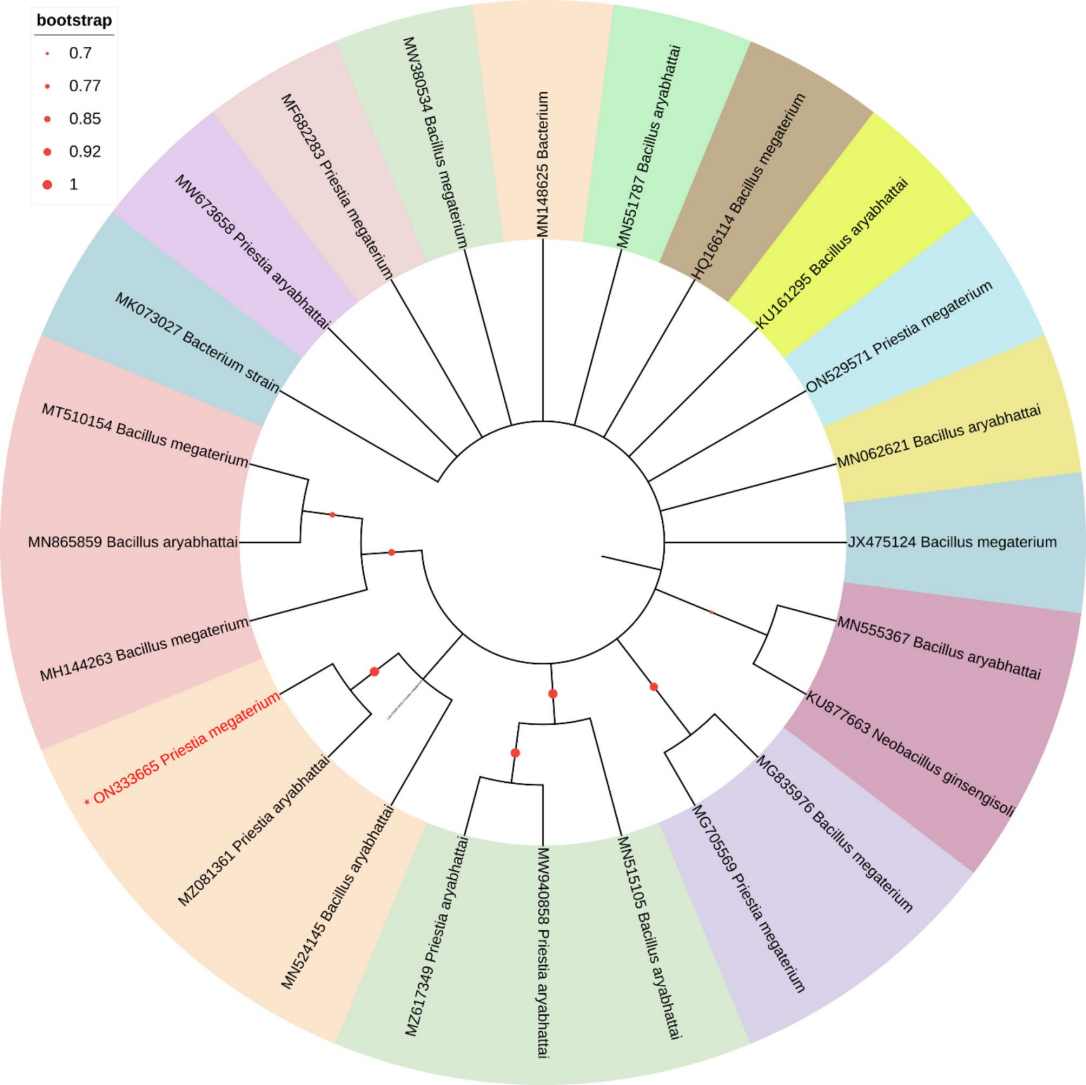
The Neighbor-Joining approach was used to infer the evolutionary history. The evolutionary history of the taxa investigated is represented by the bootstrap consensus tree, which is derived from 1000 replicates. The Maximum Composite Likelihood approach was utilized to calculate the evolutionary distances, which are expressed in the units of base substitutions per site

This analysis involved 30 nucleotide sequences. There were a total of 1510 positions in the final dataset. The phylogenetic tree analysis revealed that *Priestia magaterium* (ON333666) were clustered with Bacterium, *Priestia megaterium* and *Bacillus aryabhattai* in the phylogenetic tree (Fig. 4). This analysis involved 19 nucleotide sequences. There were a total of 1602 positions in the final dataset. The phylogenetic tree analysis revealed that, *Serratia* sp. and *Serratia marcescens* (ON337529) clustered in one group in the phylogenetic tree (Fig. 5). This analysis involved 25 nucleotide sequences. There were a total of 1520 positions in the final dataset. The phylogenetic tree analysis revealed that *Priestia magaterium* (ON333665) were clustered with *Priestia aryabhattai* and *Bacillus aryabhattai* in the phylogenetic tree (Fig. 6).

## Discussion

Pulses are cultivated worldwide and are significant source of vitamins, fiber and minerals. Among all the pulses, chickpea is one of the most important crop in India. However, its yield is substantially affected by diseases caused by various fungal pathogens. The most important soil borne and foliar pathogen infecting chickpea are *Fusarium oxysporum* f. sp. *ciceri* (Fusarium wilt) and *Botrytis cinerea* (Botrytis gray mold), respectively. Therefore, the management of the disease caused by these pathogens mostly rely on the application of chemical fungicides which have negative impact both on plant and human health. Since, the bio-agents have shown tremendous potential in managing several plant diseases, the ecofriendly management options seems to be sustainable and cheaper alternative. In the present study, the bacterial isolates were isolated from rhizosphere of healthy chickpea plants and these rhizospheric dwelling bacterial isolates were evaluated and used as potential biocontrol agents against pathogenic fungus, FOC and *B. cinerea* infecting chickpea. Several studies have also demonstrated the efficacy of these biocontrol agents in controlling many fungal pathogens of different crops. The soil samples were collected from four different regions of the Bundelkhand region of India and potential antagonistic bacteria were isolated. The morphological assay revealed that, all the 67 isolated bacteria were rod-shaped irrespective of either being gram-positive or gram-negative. All the isolated bacteria were clustered into seven different groups based on the morphological characteristics. There are several reports available on the morphological characteristics of the rhizospheric bacteria that complement the results of this study [56, 57].

The biocontrol activity of the bacterial isolates identified in this study against both the fungal pathogens was confirmed through multiple assays. In an in vitro antagonism test through dual culture assay, the isolated bacteria were found to reduce the mycelial growth of both FOC and *B. cinerea* with varying inhibition percentages. The results of the antagonistic effect of isolated bacteria revealed that, isolate 15d showed a maximum percentage of inhibition against both the fungal pathogens i.e. FOC (67.84%) and *B. cinerea* (75.29%). However, isolate 14a showed a percentage of inhibition of 67.45% and 59.22% against FOC and *B. cinerea*, respectively. Similarly, 09c has also showed a percentage of inhibition of 58.43% and 74.12% against both the fungal pathogens i.e. FOC and *B. cinerea* respectively. In the similar study, the bacterial isolates were reported to reduce *B. cinerea* growth by 75% and FOC growth by 70–75% [57].

In the present study, based on the biochemical characterization, the isolated antagonistic bacteria belong to *Enterobacteriaceae, Bacillaceae* and *Pseudomonadaceae* family. These promising bacteria were further biochemically characterized and discovered to be positive for one or more processes such the cellulose activity, ammonia, HCN, siderophore and IAA production. Almost all isolates, except three, were positive for the production of cellulase, which align with available reports where the biocontrol agents produce lytic enzymes and cellulase to limit invasion of pathogen to the plants [58]. The cellulase catalyses the hydrolysis of 1,4-D-glycosidic bonds in cellulose, which aids in the lysis of the pathogen’s cell wall [59]. The hydrolytic enzymes produced by bacteria belonging to *Pseudomonas sp.* has been shown to contribute to suppression of diseases in chickpea and green gram by inhibiting growth of phytopathogenic fungi and also promote nodulation of legumes by rhizobia [60]. Out of the three identified antagonistic bacteria, only 14a showed positive results for ammonia production. Ammonia is a recognized byproduct of some rhizospheric bacteria that function as biocontrol agent [60]. The involvement of ammonia released by *Lysobacter capsici* AZ78 in the inhibition of *Rhizoctonia solani* by the tested rhizosphere bacteria has been confirmed [61]. In the present study, no bacterial isolates were found to produce HCN. Similarly, the HCN production was not observed in the *Bacillus strains* [58, 62]. Furthermore, all the three identified isolates (15d, 9c and 14a) were found to be positive for the production of siderophores, while others were negative for the test. It has been reported that, competition for iron through the release of siderophores by antagonists reduced the mycelial growth of the pathogenic fungus *B. cinerea* infecting chickpea [63]. In another study, it was shown that, the siderophore producing rhizobacteria had a strong antagonistic effect against FOC [58].

Based on the antagonistic effect posed by the isolated bacteria, all three promising isolates were further subjected to in vivo studies to assess their potential effect under pot conditions. Similar, antagonistic effects were observed by the identified bacteria (15d, 09c and 14a) under in vivo and in vitro conditions. Similar experiments were conducted by several workers which showed the consistent results of glasshouse experiment with rhizobacteria with their in vitro studies [64] 25].

The bacterial identification has been done based on 16S rRNA gene sequence analysis [65, 66]. In this study, the 16S rRNA gene of three antagonistic bacterial isolates against FOC and *B. cinerea* of chickpea both under in vitro and in vivo conditions were sequenced and identified by BLAST homology analysis of the obtained sequences. The isolates 14a and 9c were 100% similar to *Priestia megaterium*, while the nucleotide similarity of isolate 15d was 100% to *Serratia marcescens*. *P. megaterium* has shown to possess the antagonistic ability for the management of the Fusarium wilt of chickpea [67]. Further, B2 strain of *S. marcescens* was reported to be an efficient biocontrol agent against gray mold of cyclamen caused by *B. cinerea* [28]. Furthermore, some bacterial biocontrol agents, notably *Serratia*, have been shown to engage in mutualistic interactions with plant and human hosts [25]. Therefore, assessing the risk of each potential biocontrol bacteria is equally important in order to prevent utilizing potential human pathogens as agricultural inputs.

## Conclusion

The study revealed that, the isolated rhizobacteria i.e. *P. megaterium* and *S. marcescens* have the potential to be used as biocontrol agents in the management of Fusarium wilt and BGM disease of chickpea. However, it is critical to assess the risk of each potential biocontrol bacteria to avoid using potential human pathogens as agricultural inputs, particularly with regard to agents like *Serratia* spp. where its pathogenic strains used to produce hemolysins. Therefore, it is imperative to test for hemolytic activity before its effective use as biocontrol agent. Bioformulations that combine different strains, nutrients, metabolites, and other natural products, on the other hand, must consider the compatibility of the diverse components. Further, these bioagents may be tested in conjugation with nanomaterials for enhancing the efficacy for managing the fungal diseases in chickpea. Additional efforts should be made to further validate the outcomes of the study, enhance our understanding of these bioagents, increase their performance by developing effective formulations, application methods, and integrated strategies.

### Electronic supplementary material

Below is the link to the electronic supplementary material.

**Additional file 1: Table S1** Clusters of bacterial isolates having similar morphological characters

**Additional file 2: Table S2** Biochemical characteristics of the antagonistic bacteria isolated from the rhizosphere soil samples

**Additional file 3: Table S3** Bacterial Screening for PGP factors and antimicrobial secondary metabolites production

## Data Availability

All data generated in this study are included in this article and the supplementary files. The 16S rRNA gene sequences generated for the antagonistic bacteria in the present study are available in National Center for Biotechnology Information database (*Priestia megaterium*: ON333666, ON333665and *Serratia marcescens*: ON337529).

## Abbreviations

°C: Degree Celsius
μg/ml: Microgram per milliliter
μl: Microliter
BOD: Biological Oxygen Demand
CMC: Carboxymethyl Cellulose
CTAB: Cetyltrimethylammonium Bromide
Fo: *Fusarium oxysporum*
g/l: Gram per liter
HCN: Hydrogen Cyanide Production
ICAR: Indian Council of Agricultural Research
KOH: Potassium Hydroxide
mm: Millimeter
NaCl: Sodium Chloride
