# Supplementary material for: Evaluation of biocontrol efficacy of rhizosphere dwelling bacteria for management of Fusarium wilt and Botrytis gray mold of chickpea
Source: BMC Genom Data. 2024 Jan 15;25:7. doi: 10.1186/s12863-023-01178-7 (PMC10790480; doi:10.1186/s12863-023-01178-7)
Supplement: Supplementary file 1 — Additional file 1: Table S1 Clusters of bacterial isolates having similar morphological characters [file 12863_2023_1178_MOESM1_ESM.docx]

**Table S1** Clusters of bacterial isolates having similar morphological characters

| **Cluster** | **Isolate** | **Shape** | **Colour** | **Margins** | **Opacity** | **Appearance** | **Gram stain** | **Cell shape** |
| --- | --- | --- | --- | --- | --- | --- | --- | --- |
| I | 6b, 6d, 10a, 13b, 18c,  21b, 22d, 26d, 6c, 36a | round / oval | yellow | smooth | opaque/ semi transparent | shiny/dull | Pink | Rod |
| II | 10c, 12c, 14c, 18a, 22a, 27c, 8a, 19a | round/ oval | cream | smooth | opaque/ semi transparent | shiny | Blue | Rod |
| III | 15a, 1a, 9d,16c,18e, 21a, 22e, 26a, 26b, 27b, 37b, 15c, 35b, 38a | round/ oval | cream | smooth | opaque/ semi transparent | shiny/dull | Pink | Rod |
| IV | 12b, 15d, 27d, 40c, 10e, 37a, 16a, 33a, 10b, 40a, 40b | round | white/ off white | smooth | opaque/ semi transparent | shiny/dull | Blue | Rod |
| V | 8b, 8c, 27a, 36b, 14b, 22b, 10d | round/ irregular | white/ off white | serrate/ irregular | opaque/ semi transparent | shiny/dull | Blue | Rod |
| VI | 35a, 22c, 9a, 26c, 12a, 18d, 21c, 9b, 15b, 16b | round | white/ off white | smooth | semi transparent | shiny/dull | Pink | Rod |
| VII | 9c, 6a, 14a, 3a, 13a, 18b, 19b | round/ irregular | white/ off white | rhizoid/ irregular | semi transparent | shiny/dull | Pink | Rod |
