# Supplementary material for: Evaluation of biocontrol efficacy of rhizosphere dwelling bacteria for management of Fusarium wilt and Botrytis gray mold of chickpea
Source: BMC Genom Data. 2024 Jan 15;25:7. doi: 10.1186/s12863-023-01178-7 (PMC10790480; doi:10.1186/s12863-023-01178-7)
Supplement: Supplementary file 2 — Additional file 2: Table S2 Biochemical characteristics of the antagonistic bacteria isolated from the rhizosphere soil samples [file 12863_2023_1178_MOESM2_ESM.docx]

**Table S2** Biochemical characteristics of the antagonistic bacteria isolated from the rhizosphere soil samples

| **Sl. No.** | **Isolate** | **KOH** | **A** | **I** | **Ci** | **M.R.** | **V.P.** | **M** | **Ca** | **H_2_S** | **O** | **Bacterial family** |
| --- | --- | --- | --- | --- | --- | --- | --- | --- | --- | --- | --- | --- |
|  | 6a | + | - | - | - | - | - | + | + | + | + | *Pseudomonadaceae* |
|  | 8b | - | + | - | - | - | - | - | + | + | + | *Bacillaceae* |
|  | 8c | - | + | - | - | - | + | + | + | + | + | *Bacillaceae* |
|  | 9c | + | - | - | + | - | - | + | + | + | + | *Pseudomonadaceae* |
|  | 10b | - | + | - | - | - | - | - | + | + | + | *Bacillaceae* |
|  | 10c | - | + | - | - | - | - | + | + | + | - | *Bacillaceae* |
|  | 14a | + | - | - | + | - | + | + | + | + | + | *Pseudomonadaceae* |
|  | 15c | + | - | - | + | - | - | + | + | + | - | *Pseudomonadaceae* |
|  | 15d | - | + | - | + | - | - | + | + | + | + | *Enterobacteriaceae* |
|  | 22a | - | + | - | - | - | - | + | + | + | - | *Bacillaceae* |
|  | 40a | - | + | - | + | - | - | - | + | + | - | *Bacillaceae* |
|  | 40b | - | - | - | - | - | - | + | + | + | + | *Bacillaceae* |
|  | 40c | - | + | - | - | - | - | - | + | + | - | *Bacillaceae* |

KOH-Potassium hydroxide; A-amylase; I-Indole; Ci-Citrate; M.R.-Methyl Red; V.P.-Voges Proskauer’s; M-Motility; Ca-Catalase; H_2_S-Hydrogen sulfide; O-Oxidase; ‘+’ positive; ‘− ’ negative
