# Supplementary material for: Evaluation of biocontrol efficacy of rhizosphere dwelling bacteria for management of Fusarium wilt and Botrytis gray mold of chickpea
Source: BMC Genom Data. 2024 Jan 15;25:7. doi: 10.1186/s12863-023-01178-7 (PMC10790480; doi:10.1186/s12863-023-01178-7)
Supplement: Supplementary file 3 — Additional file 3: Table S3 Bacterial Screening for PGP factors and antimicrobial secondary metabolites production [file 12863_2023_1178_MOESM3_ESM.docx]

**Table S3** Bacterial Screening for PGP factors and antimicrobial secondary metabolites production

| **Sl. No.** | **Isolate** | **Cellulase** | **Ammonia** | **Siderophore** | **IAA** | **HCN** |
| --- | --- | --- | --- | --- | --- | --- |
|  | 6a | - | - | ++ | +++ | - |
|  | 08b | +++ | + | - | ++ | - |
|  | 08c | +++ | + | - | + | - |
|  | 09c | ++ | - | ++ | ++ | - |
|  | 10b | ++ | - | - | ++ | - |
|  | 10c | ++ | ++ | - | +++ | - |
|  | 14a | + | ++ | ++ | - | - |
|  | 15c | +++ | - | - | +++ | - |
|  | 15d | +++ | - | ++ | ++ | - |
|  | 22a | - | - | - | ++ | - |
|  | 40a | ++ | - | - | +++ | - |
|  | 40b | - | - | - | +++ | - |
|  | 40c | +++ | - | - | ++ | - |

(-) no activity; (+) low; (++) moderate; (+++) high
